# Inclusion of Platinum Agents in Neoadjuvant Chemotherapy Regimens for Triple-Negative Breast Cancer Patients: Development of GRADE (Grades of Recommendation, Assessment, Development and Evaluation) Recommendation by the Italian Association of Medical Oncology (AIOM)

**DOI:** 10.3390/cancers11081137

**Published:** 2019-08-08

**Authors:** Maria Vittoria Dieci, Lucia Del Mastro, Michela Cinquini, Filippo Montemurro, Laura Biganzoli, Laura Cortesi, Alberto Zambelli, Carmen Criscitiello, Alessia Levaggi, Benedetta Conte, Massimo Calabrese, Alba Fiorentino, Caterina Marchiò, Corrado Tinterri, Veronica Andrea Fittipaldo, Giovanni Pappagallo, Stefania Gori

**Affiliations:** 1 Department of Surgery, Oncology and Gastroenterology, University of Padova, via Giustiniani 2, 35128 Padova, Italy; 2 Medical Oncology 2, istituto Oncologico Veneto IRCCS, via Gattamelata 64, 35128 Padova, Italy; 3 Department of Internal Medicine and Medical Specialties (DIMI), School of Medicine, University of Genova, viale Benedetto XV 6, 16132 Genova, Italy; 4 Department of Medical Oncology, UO Oncologia Medica 2, Policlinico San Martino-IST, Largo Rosanna Benzi 10, 16132 Genova, Italy; 5 Oncology Department, Mario Negri Institute for Pharmacological Research IRCCS, via Giuseppe La Masa 19, 20156 Milano, Italy; 6 Day Hospital Oncologico Multidisciplinare, Istituto di Candiolo, FPO-IRCCS, SP 142 Km3.95, 10060 Candiolo, Torino, Italy; 7 “Sandro Pitigliani” Medical Oncology Department, Hospital of Prato, Via Suor Niccolina Infermiera 20, 59100 Prato, Italy; 8 Department of Oncology and Hematology, University Hospital of Modena, via del Pozzo 71, 41124 Modena, Italy; 9 Medical Oncology, Azienda Ospedaliera Papa Giovanni XXIII, Piazza OMS 1, 24127 Bergamo, Italy; 10 Division of Early Drug Development, European Institute of Oncology, via Ripamonti 435, 20141 Milano, Italy; 11 Department of Oncology, Sant’Andrea Hospital, Via Vittorio Veneto 197, 19121 La Spezia, Italy; 12 Breast Radiology, IRCCS-Policlinico San Martino, Largo Rosanna Benzi 10, 16132 Genova, Italy; 13 Radiation Oncology Department, General Regional Hospital “F. Miulli”, Strada Provinciale 127, 70021 Acquaviva delle Fonti (Bari), Italy; 14 FPO-IRCCS Candiolo Cancer Institute, SP 142 Km3.95, 10060 Candiolo, Italy; 15 Department of Medical Sciences, University of Turin, via Verdi 8, 10124 Turin, Italy; 16 Department of Surgery, IRCCS Clinical and Research Institute Humanitas, Via Manzoni 56, 20089 Rozzano, MI, Italy; 17 Epidemiology & Clinical Trials Office, General Hospital, Via Don Giacobbe Sartor 4, 30035 Mirano, VE, Italy; 18 Medical Oncology Unit, Sacro Cuore—Don Calabria Hospital, Cancer Care Center, Via Don Angelo Sempreboni 5, 37024 Negrar, VR, Italy

**Keywords:** triple-negative breast cancer, neoadjuvant chemotherapy, platinum, GRADE methodology, clinical recommendation

## Abstract

In the absence of identified therapeutic targets, chemotherapy is the main systemic treatment option for triple-negative breast cancer (TNBC). The achievement of a pathological complete response (pCR) after neoadjuvant chemotherapy leads to good outcome, whereas patients not achieving a pCR are at high risk of relapse. Various trials have evaluated the inclusion of platinum in neoadjuvant chemotherapy regimens for TNBC, leading to non-univocal results. The panel of the Italian Association of Medical Oncology (AIOM) Guidelines on Breast Cancer developed a clinical recommendation on the addition of platinum to anthracycline/taxane-based neoadjuvant chemotherapy for TNBC by using the Grades of Recommendation, Assessment, Development, and Evaluation (GRADE) methodology and the Evidence to Decision framework (EtD). Five studies were eligible. The panel identified the following outcomes of benefit: pCR (critical), disease/event-free survival (DFS/EFS, critical), and overall survival (OS, critical). The panel identified febrile neutropenia (critical), serious adverse events (critical), anemia grade 3–4 (important), thrombocytopenia grade 3–4 (important) as outcomes of harms. The probability of pCR was higher in the platinum-based chemotherapy group versus control group (RR = 1.45, 95%CI 1.28–1.64); however, no impact on long-term outcome was observed. Neoadjuvant treatment regimens containing platinum resulted in a non-significant increase in the risk of febrile neutropenia and in a significant increase in the risk serious adverse events, G3–G4 anemia and G3–G4 thrombocytopenia: 11.3% versus 0.8%, RR = 15.66 (95%CI 6.38–38.44). The panel judged uncertain/favorable the benefit/harms balance. The panel’s final recommendation was conditional in favor of the inclusion of platinum in anthracycline/taxane-based neoadjuvant regimens for TNBC.

## 1. Introduction

Triple-negative breast cancer (TNBC) is the most aggressive breast cancer (BC) subtype, accounting for around 15% of all diagnoses. Up to one-third of patients diagnosed with TNBC may experience disease recurrence, with the highest peak within 1–3 years from diagnosis. The pattern of recurrence often involves visceral sites, leading to high mortality rates and short survival. Triple-negative breast cancer lacks recognized therapeutic targets; therefore, cytotoxic agents represent the only option of systemic treatment [1,2].

Despite its aggressiveness, TNBC is highly chemosensitive [3]. This paradox is particularly evident in the neoadjuvant setting [4]. Indeed, TNBC is associated with higher pathological complete response (pCR) rates (~30–40%) after neoadjuvant chemotherapy (NACT) as compared to other BC subtypes. Moreover, the achievement of pCR has the strongest prognostic effect for TNBC as compared to other subtypes: TNBC patients who achieve a pCR have an excellent survival, on the other hand, among patients who fail to achieve a pCR, those with TNBC show the highest risk of relapse [4,5]. Neoadjuvant chemotherapy is the standard of care for locally advanced and inflammatory disease but is also widely used for most of the patients with operable TNBC. Based on the response achieved after NACT, treatment for TNBC may be further modulated. For example, there is the opportunity to de-escalate local treatment in case of good response (i.e., convert from mastectomy to conservative surgery) or to escalate systemic treatment (i.e., further adjuvant chemotherapy or inclusion in adjuvant clinical trials) for patients not achieving a pCR [6,7].

Triple-negative breast cancer represents the vast majority of *BRCA1*-related BCs (67.5%), whereas about 7.5% of *BRCA2*-related BCs are TNBCs [8,9]. Even if the majority of all TNBCs arise in the absence of a *BRCA* germline mutation, these tumors often present features of DNA repair deficiency. These considerations have led to hypothesize that TNBC may be particularly susceptible to DNA-damaging agents [1]. Platinum-containing NACT have been tested in TNBC with the aim to increase the proportion of patients achieving a pCR and ultimately to improve long-term outcome, leading to mixed results [10,11]. Current recommendations and statements in main international guidelines are controversial:

- National Comprehensive Cancer Network (NCCN) Breast Cancer Guidelines, version 1.2019 [12]: “…the NCCN panel does not recommend addition of carboplatin to neoadjuvant standard chemotherapy for patients with triple-negative breast cancer outside a clinical trial setting”;
- Early breast cancer: European Society of Medical Oncology (ESMO) Clinical Practice Guidelines for diagnosis, treatment and follow up (2019) [13]: “The addition of a platinum compound may be considered in triple-negative tumors and/or in patients with deleterious *BRCA1/2* mutations [I, C]” (statement related to neoadjuvant chemotherapy);
- St. Gallen Consensus Conference 2017 [14]: “The Panel clearly recommended against routine use of platinum-based chemotherapy in unselected TNBC cases. In *BRCA1/2* associated cancers, the Panel was evenly split on whether to recommend (neo)adjuvant platinum chemotherapy though agreed that such patients should receive alkylating chemotherapy in addition to a taxane and anthracycline”;
- European School of Oncology (ESO)—ESMO consensus guidelines for BC in young women (BCY3) [15]: “In patients with TNBC or BRCA-associated tumors the incorporation of platinum agents increases pCR rates and may be considered when neoadjuvant chemotherapy is indicated. Data on the impact of incremental increases in pCR on long term outcome are not conclusive. The use of platinum derivatives has potential additional impact on fertility and increased toxicity that may compromise standard duration and dosing of systemic treatment, and this needs to be clearly communicated to patients”, Level of Evidence IIA (weak recommendation, high-quality evidence), 77% consensus.

None of these recommendations were developed with the Grades of Recommendation, Assessment, Development, and Evaluation (GRADE) approach [16].

The panel of the Italian Association of Medical Oncology (AIOM) Guidelines on Breast Cancer decided to develop in 2018 a clinical recommendation by applying the GRADE approach to address the clinical question related to the inclusion of platinum in NACT for TNBC.

## 2. Results

### 2.1. Search Strategy Results and Details of the Identified Relevant Studies

**Figure 1:**
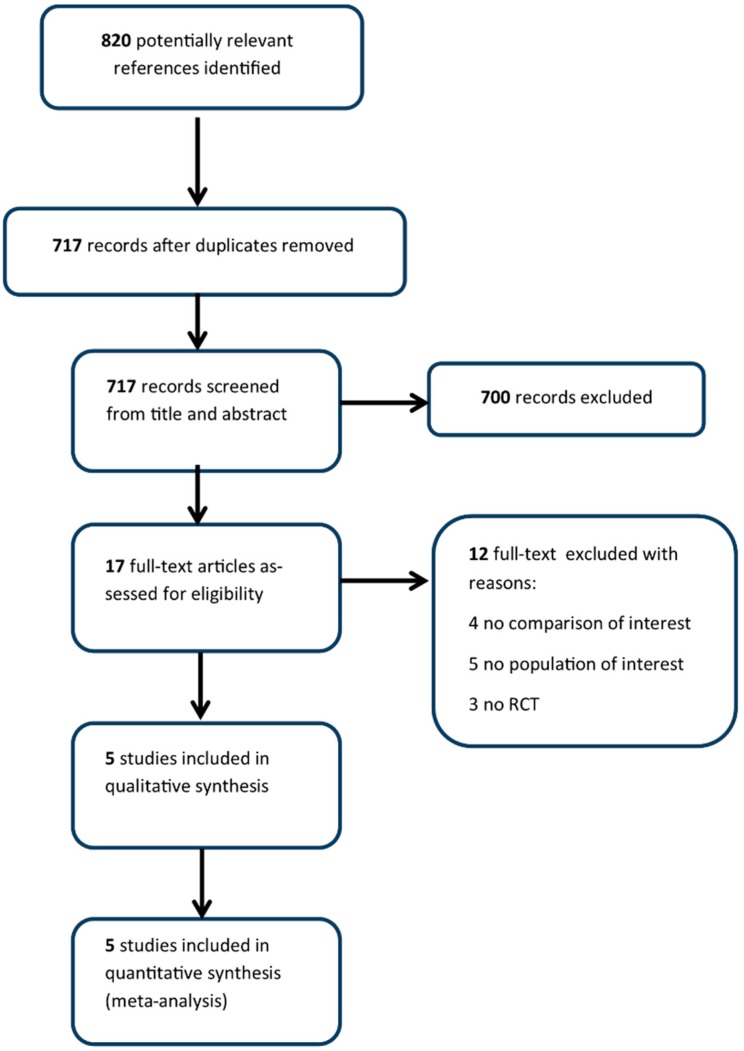
PRISMA (Preferred Reporting Items for Systematic Reviews and Meta-Analyses) flow chart.

The literature search returned 820 records. Of the 17 full-text articles assessed for eligibility, 5 studies [17,18,19,20,21] met the eligibility criteria (Figure 1, Table 1).

The BrighTNess study is a phase III randomized trial that randomized patients with TNBC (estrogen and progesterone receptor <1% and HER2 negative) to receive: paclitaxel + carboplatin + veliparib followed by doxorubicin and cyclophosphamide; paclitaxel + carboplatin + veliparib placebo followed by doxorubicin and cyclophosphamide; paclitaxel + carboplatin placebo + veliparib placebo followed by doxorubicin and cyclophosphamide [17]. Eligibility criteria included: stage II–III disease and documented germline *BRCA* test available. The primary endpoint was pCR (ypT0/is ypN0; assessed by local pathologist), secondary endpoints included event-free survival, overall survival, and breast-conserving surgery. For the primary endpoint analysis, the prespecified pairwise comparisons were: paclitaxel + carboplatin + veliparib versus paclitaxel + carboplatin and paclitaxel + carboplatin + veliparib versus paclitaxel. Between 2014 and 2016, a total of *n* = 634 patients were randomized (2:1:1; *n* = 316 paclitaxel + carboplatin + veliparib; *n* = 160 paclitaxel + carboplatin; *n* = 158 paclitaxel). Randomization was stratified according to germline *BRCA* mutation, nodal stage (N0 versus N1–2) and planned schedule of doxorubicin and cyclophosphamide (every 2 or 3 weeks). The population included: 50% of patients aged ≤ 50 years, 15% of patients with a *BRCA* mutation, 83% of patients with T1 or T2, 58% of patients with clinical node negative stage. For 55%, the planned schedule of doxorubicin and cyclophosphamide administration was every 2 weeks.

The CALGB 40603 is a phase II study that enrolled 443 patients with stage II or III non-inflammatory TNBC (estrogen and progesterone receptor ≤ 10% and HER2 negative) [18]. Patients received paclitaxel followed by dose-dense doxorubicin + cyclophosphamide. Patients were randomized in a 2 × 2 design to receive in addition carboplatin and/or bevacizumab (10 mg/kg every 2 weeks for 9 courses concurrently to paclitaxel and the first 3 cycles of doxorubicin and cyclophosphamide). Randomization was stratified by stage (II or III). The primary endpoint was pCR in the breast (ypT0/is, assessed by local pathology). Two separate pairwise comparisons were planned: carboplatin (+/− bevacizumab) versus no carboplatin (+/− bevacizumab) and bevacizumab (+/− carboplatin) versus no bevacizumab (+/− carboplatin). Secondary endpoints included: pCR in breast and axilla (ypT0/is ypN0), treatment delivery, treatment-related toxicities, residual cancer burden, conversion from node-positive to pathologically node-negative status, conversion to breast-conserving surgery. Patients were also monitored for relapse-free survival, time to first failure and overall survival. From 2009 to 2012, 454 patients were enrolled: 60% aged 40–59 years, 68% clinical stage II, 42% N0.

The GeparSixto phase II randomized trial compared the following neoadjuvant treatments: paclitaxel + non-pegylated liposomal doxorubicin (versus the same regimen combined with concomitant carboplatin [19]. Patients diagnosed with TNBC (estrogen and progesterone receptor <1% and HER2 negative) or HER2-positive BC were eligible. Triple negative breast cancer patients also received concomitant bevacizumab 15 mg/kg every 3 weeks; HER2-positive patients also received concomitant trastuzumab (8 mg/kg loading dose followed by 6 mg/kg every 3 weeks) and lapatinib (750 mg daily). Patients were eligible if they had clinical stage T2 to T4a–d tumors or T1c tumors with clinical or histological node-positive disease. The randomization (1:1) was stratified by: biological subtype (TNBC, HER2-positive/hormone receptor-negative, HER2-positive/hormone receptor positive) and Ki67 level (≤20% or >20%). The primary endpoint was pCR (ypT0/ypN0) assessed by local pathologist (pathology reports reviewed by an independent pathologist). Secondary outcomes were: tolerability, treatment adherence, clinical/radiological response rates, pathological stages ypT0/is ypN0, ypT0/is ypN0/+, and any ypT ypN0, regression grade, rate of breast, and axilla conservation. Efficacy was assessed in pre-defined subgroups according to central triple-negative and HER2-positive subtypes and Ki67 levels. From 2011 and 2012, 588 patients were enrolled (*n* = 293 without carboplatin, *n* = 295 with carboplatin): the majority had cT2 stage (64%) or cN0 stage (53%) by ultrasonography, *n* = 315 patients had TNBC.

The GEICAM/2006-03 phase II study enrolled patients from 2007 to 2010. Key eligibility criteria were: basal-like BC (estrogen and progesterone receptor < 1%, HER2-negative, cytokeratin 5/6 or epidermal growth factor receptor positive by immunohistochemistry), tumor size > 2 cm or <2 cm with nodal involvement [20]. Patients were randomized 1:1 to receive: epirubicin + cyclophosphamide followed by docetaxel or to epirubicin + cyclophosphamide followed by docetaxel + carboplatin. Randomization was stratified according to institution, tumor size, histological tumor grade, and axillary status. The primary endpoint was pCR in the breast. Secondary endpoints were: safety, clinical response, breast conservative surgery, and axillary node status at the time of surgery. Although not formally included as a study endpoint, the authors have also reported pCR in breast and axilla. A total of 94 patients were enrolled: *n* = 46 received treatment without platinum, *n* = 47 received treatment with platinum, *n* = 1 never started treatment. Median age was 47 years, 69% of patients had cT2 stage and 48% were node-negative at baseline.

The UMIN000003355 study is a phase II randomized trial for patients with HER2-negative BC [21]. Patients were eligible if they had a tumor larger than 2 cm or a smaller tumor with clinically positive axillary nodes (stages II-IIIA). Patients with T4 or N3 were excluded. Between 2010 and 2011, a total of 181 patients entered the study, two of which refused to undergo treatment. Patients were randomized to receive paclitaxel 80 mg/mq weekly for 12 weeks followed by cyclophosphamide + epirubicin + 5-fluorouracil (500 mg/mq, 100 mg/mq, 500 mg/mq every 3 weeks for 4 courses) or to the same regimen with the addition of Carboplatin AUC 5 every 3 weeks for 4 courses administered concomitantly to paclitaxel. Randomization was balanced for stage (II versus IIIA), hormone receptor status and institution. The primary endpoint was pCR (ypT0/is ypN0), evaluated by central pathology. Secondary endpoints were: disease-free survival, clinical response rate, breast conservation rate, safety. Median age in the intention-to-treat population was 47 years, 82% of patients had stage II disease, 35% had node negative status, 42% were triple negative (*n* = 75; *n* = 37 with carboplatin, *n* = 38 without carboplatin).

### 2.2. Outcomes of Benefit

Table 2 reports a summary of certainty of evidence assessment, absolute and relative effects (evidence profile table).

#### 2.2.1. pCR (ypT0/is ypN0)

All the five identified studies reported pCR rates [17,18,19,20,21]. For GeparSixto [19] and UMIN000003355 [21] studies, only data for TNBC patients were considered. With regards to the CALGB 40603 study, data from the carboplatin arms (+/− bevacizumab) and the no-carboplatin arms (+/− bevacizumab) were included, in accordance with the 2 × 2 design [18]. The results of the paclitaxel + carboplatin + veliparib placebo and the paclitaxel + carboplatin placebo + veliparib placebo arms were considered for the BrighTNess study, in order to avoid potential confounding effect of veliparib [17].

The certainty of evidence was judged as “low” due to the possible detection bias (lack of blinded outcome assessor in most of the studies, with the exception of BrighTNess) and results heterogeneity (I-squared = 55%). Overall, the probability of pCR was of 54 in every 100 patients in the platinum-based chemotherapy group and 38 in the control group (RR = 1.45, 95%CI 1.28–1.64).

#### 2.2.2. DFS/EFS and OS

Four studies included DFS/EFS as secondary endpoint [17,18,19,21], but only two reported survival data: CALGB 40603 [22] and GeparSixto [23,24] (TNBC subgroup considered). The certainty of evidence was judged as “very low” for DFS/EFS for the following reasons: heterogeneity (I-squared = 63.9%) and imprecision (low number of events). Moreover, a possible publication bias was detected. Indeed, both the BrighTNess [17] and the UMIN0000035 [21] trials had DFS/EFS has secondary endpoints but have not reported data yet. With regards to BrighTNess, the follow-up period may be too short to conduct such type of analysis; therefore, no publication bias was suspected. On the other hand, seven years have lasted since the last patient enrollment in the UMIN000003355 trial, but survival data have never been reported. No significant difference was observed for the comparison of platinum versus non-platinum containing regimens (HR = 0.72, 95%CI 0.49–1.06).

Three studies included OS as secondary endpoint [17,18,19], but only two reported survival data: CALGB 40603 [22] and Geparsixto [23,24] (TNBC subgroup considered). The certainty of evidence was judged as “low” for OS for the following reasons: lack of blinding in GeparSixto, heterogeneity (I-squared = 33%), and imprecision (low number of events). No publication bias was suspected for BrighTNess since the follow-up period was probably too short to report OS analysis. No significant OS difference was observed for the comparison of platinum vs. non-platinum containing regimens (HR = 0.86, 95%CI 0.46–1.63).

### 2.3. Outcomes of Harm

Table 2 reports a summary of certainty of evidence assessment, absolute and relative effects (evidence profile table).

All five studies reported data for febrile neutropenia (critical), thrombocytopenia G3–4 (important) and anemia G3–4 (important) [17,18,19,20,21]. Three studies [17,18,19] (BrighTNess, CALGB40603 and GeparSixto) reported the rate of serious adverse events (critical).

For CALGB 40603 study, only the arms without bevacizumab were considered, since the addition of the antiangiogenic drug had some effect on safety [18].

From the BrighTNess trial, safety data of the paclitaxel + carboplatin + veliparib placebo and paclitaxel + carboplatin placebo + veliparib placebo were considered, in order to avoid potential confounding from the administration of veliparib [17]. The authors of the BrighTNess study reported the safety data separately for the first and second segments of therapy (taxane-based and doxorubicin/cyclophosphamide, respectively) [17], and it was not possible to extrapolate from the publication the overall rate of specific adverse events occurring in the entire treatment period since the same patients might have experienced the same event in the first and second segment. The panel opted to consider in the evidence profile table the events occurring in the first segment, but also discussed the rate of events described for the second segment and took into consideration in the assessment process. This was based on the assumption that the previous exposure to taxane-based regimens with potentially different toxicity profile could influence the tolerability of subsequent anthracycline chemotherapy.

In the GeparSixto [19] and UMIN00000335 [21] trials, safety data were reported for the entire population of patients and not according to tumor biology. However, the results of these trials were included in the analysis, assuming that HER2 and/or hormone receptor status should have no effect on safety.

#### 2.3.1. Febrile Neutropenia

The certainty of evidence was judged as “moderate” due to the imprecision (low number of events). Neoadjuvant treatment regimens containing platinum resulted in a non-significant increase in the risk of febrile neutropenia: 9% versus 6.3%, RR = 1.42 (95%CI 0.98–2.06).

In the BrighTNess study, 26 patients treated with carboplatin and 7 treated without carboplatin experienced a febrile neutropenia in the second segment of study treatment (16.5% and 4.5%) [17].

#### 2.3.2. Serious Adverse Events

The certainty of evidence was judged as “moderate” due to the lack of masking in the CALGB 40,603 trial. The addition of platinum to an anthracycline and taxane-based chemotherapy regimen resulted in significantly higher rate of serious adverse events: 30.7% versus 24.0%, RR = 1.28 (95%CI 1.06–1.55).

In the BrighTNess study, 32 and 16 SAEs were reported in the second treatment segment for patients treated with carboplatin and patients not treated with carboplatin (20.3% and 10.2%) [17].

#### 2.3.3. Anemia G3–G4

The certainty of evidence was judged as “moderate” due to the imprecision (low number of events). Neoadjuvant treatment regimens containing platinum resulted in a significantly higher risk of G3–G4 anemia: 14.1% versus 0.3%, RR = 49.08 (95%CI 12.15–198.20).

In the BrighTNess study, 36 patients treated with carboplatin and 12 treated without carboplatin experienced G3–G4 anemia in the second segment of study treatment (22.8% and 7.6%) [17].

#### 2.3.4. Thrombocytopenia G3–G4

The certainty of evidence was judged as “moderate” due to the imprecision (low number of events). The addition to platinum to an anthracycline and taxane-based chemotherapy regimen resulted in significantly higher risk of G3–G4 thrombocytopenia: 11.3% versus 0.8%, RR = 15.66 (95%CI 6.38–38.44).

In the BrighTNess study, 10 patients treated with carboplatin and two treated without carboplatin experienced a G3–G4 thrombocytopenia in the second segment of study treatment (6.3% and 1.3%) [17].

### 2.4. EtD (Evidence to Decision) Framework

The full EtD table is reported in Table 2.

In summary, the panel judged the problem addressed by the clinical question as a priority, by acknowledging the unmet clinical need of improving the outcome for TNBC, the strong prognostic value of pCR.

The panel acknowledged the absence of a clear positive impact on survival by the addition of platinum. However, the observed difference in pCR favoring the addition of a platinum agent was considered sufficient to judge as “moderate” the substantiality of the desirable anticipated effects. The panel considered in this evaluation the lack of power of neoadjuvant studies to detect significant survival differences.

The panel judged as “small” the substantiality of undesirable effects. In their appraisal, panel members stated that the heterogeneity of treatment schedules across the studies [17,18,19,20,21] might have partly influenced the rate of the undesirable effects, making an overall judgement more complex. In particular, the panel noted that in most of the studies carboplatin was administered every 3 weeks, whereas, based on panel members’ opinion and experience, the most manageable schedule is carboplatin AUC every 2 weeks.

The certainty of evidence was described as very low since most outcomes were affected by inconsistency and imprecision of estimates.

### 2.5. Benefit/Harm Balance and Final Recommendation

The panel voted for the benefit/harm benefit as uncertain–favorable (10 votes out of 11 members). The strength of the recommendation was voted as conditionally positive by 10 out of 11 panel members.

Hence, the final recommendation released by the panel was:

“In patients with triple-negative breast cancer who are candidates to receive neoadjuvant chemotherapy, the addition of platinum to a standard regimen containing anthracycline and taxane may be taken into consideration”.

The final recommendation and summary of the GRADE evaluation is reported in Table 3.

## 3. Discussion

The AIOM Clinical Practice Guidelines on Breast Cancer panel suggests the addition of platinum to an anthracycline and taxane neoadjuvant regimen as an option that may be taken into consideration for TNBC patients. This recommendation derived from a critical appraisal of available evidence through a rigorous methodology and discussion, as highlighted in this paper.

Of the outcomes of benefit, only pCR rate was significantly improved with the addition of platinum, with an absolute increase of 17%. Although there was no clear benefit in survival outcomes and the analysis of harms showed a significant increase in the rate of SAEs, G3–G4 anemia and thrombocytopenia and a numerical increase in febrile neutropenia, the overall recommendation was conditional in favor of the intervention. The appraisal that led to this recommendation has been described in details in this paper. The final recommendation highlights the relevance that was attributed by the panel to the effect on pCR, which has the strongest association with long-term outcome in the challenging and hard-to-treat TNBC subtype. However, it remains unclear whether platinum use in neoadjuvant setting could also improve long-term survival, more data and longer follow up are needed. Moreover, a deeper understanding of molecular heterogeneity of TNBC and how it may guide treatment choice represent a research priority [25,26]. Some additional specific issues may deserve some further discussion.

The clinical question and the related recommendation were not restricted to *BRCA* mutation carriers only, but involved all unselected TNBC patients. In the metastatic setting, there is evidence from a randomized phase III trial that carboplatin is more effective than docetaxel as first line therapy for TNBC patients only in the subgroup with a germline *BRCA* mutation [27]. Although it was initially suggested that the benefit of neoadjuvant platinum agents could be restricted to *BRCA* carriers [28,29], this issue remains controversial [23]. Indeed, data from a recent meta-analysis suggest no differential benefit from platinum-based NACT according to *BRCA* status: pCR rates were 58.0% with platinum and 54.3% without platinum in BRCA-mutated patients and 57.0% with platinum and 32.7% without platinum in *BRCA*-wilde type patients [11]. Another meta-analysis compared the pCR rates after neoadjuvant chemotherapy with platinum compounds for *BRCA*-mutated versus non-*BRCA*-mutated TNBC patients: although the pCR rate was numerically higher for BRCA-mutated cases (58.4% versus 50.7%), the difference was not statistically significant [30]. These data may indeed highlight a higher general chemosensitivity of *BRCA*-mutated patients [31].

Alkylating agents are other fundamental drugs for TNBC, due to the mechanism of action [32,33]. In the clinical question developed by the AIOM panel, the treatment of reference is an anthracycline and taxane-containing regimen. All the studies included in the evaluation of the clinical question also included cyclophosphamide in the chemotherapy backbone [17,18,20,21], with the exception of GeparSixto [19]. This difference, together with other sources of treatment heterogeneity across studies, might have influenced the evaluation of the effects of the addition of platinum. It has to be specified that all the anthracycline and taxane-based adjuvant/neoadjuvant regimens listed in the AIOM Clinical Practice Guidelines on Breast Cancer [34] also contain cyclophosphamide. Therefore, the AIOM recommendation should be interpreted within this frame.

The selection of the outcome of harms highlighted a particular concern about hematologic toxicity, which is a typical set of adverse events related to platinum agents. Peripheral neuropathy, which is also specifically related to platinum [35], was not considered as a relevant outcome of harm. The panel commented that potential neurotoxic combinations of platinum and taxanes are already widely used in other diseases in clinical practice, with known toxicity rates. What was unique to this clinical question was the addition of platinum to a regimen containing both taxane and anthracycline, raising a major concern for myeloid suppression. Moreover, a recent meta-analysis reported low and similar rates of grade 3–4 neuropathy in platinum-containing and platinum-free neoadjuvant regimens for TNBC (3.6% in both groups) [11].

## 4. Materials and Methods

### 4.1. The AIOM Guidelines on Breast Cancer Panel

The AIOM Guidelines on Breast Cancer are updated every year by a panel composed of academics and clinicians with expertise in medical oncology, surgery, radiation therapy, pathology, radiology, and clinical research methodology. The draft of the updated guidelines is sent to external reviewers prior to the final publication on the AIOM website [34]. The external reviewers are nominated by AIOM and other relevant scientific societies (for 2018: Italian National Association of Breast Surgeons (ANISC), Italian Society of Anatomic Pathology and Diagnostic Cytopathology (SIAPEC), Italian Society of Radiation Oncology (AIRO)).

### 4.2. Development of Clinical Question

The clinical question was developed according to the P.I.C.O. acronym requiring the definition of: population (P), intervention (I), comparison (C), and outcomes (O).

For the 2018 version of the AIOM Guidelines on Breast Cancer, the panel decided to address the following clinical question:

- In patients with triple-negative breast cancer candidate to receive neoadjuvant chemotherapy, is the addition of a platinum agent to a taxane and anthracycline-containing regimen recommendable versus a taxane and anthracycline-containing regimen only?

The panel defined, as population of interest, unselected TNBC patient candidates to receive NACT without restricting to BRCA-carriers only, consistently with the approach followed by other BC guidelines. The panel considered in the clinical question an anthracycline and taxane-containing regimen as the standard reference chemotherapy, since this is the preferred treatment recommended by AIOM and other BC guidelines for TNBC [12,13,14]. Therefore, the intervention is the addition of platinum to a regimen already containing anthracycline and taxane.

### 4.3. Identification of Outcomes

The panel identified the following outcomes of benefit: pCR rate (absence of residual invasive cancer cells in both breast and axilla, ypT0/is ypN0), disease/event-free survival (DFS/EFS), and overall survival (OS). All these outcomes were judged as “critical” for the decision-making.

The panel identified the following outcomes of harm: febrile neutropenia, anemia (grade 3–4), thrombocytopenia (grade 3–4), serious adverse events (SAEs). Febrile neutropenia and SAEs were judged as “critical”, the other outcomes as “important”.

### 4.4. Search Strategy and Selection of Evidence

A systematic literature search was performed searching PubMed, Embase and Cochrane Library without date restriction up to May 2018. The full search strategy is available in Appendix A. Main articles were cross-referenced to check that all the relevant literature was fully identified. The PRISMA flow-chart is reported in Figure 1. Retrospective or prospective non-randomized studies were excluded. Studies that also enrolled non-TNBC patients were considered, provided that at least efficacy data in the subgroup of TNBC were reported. Only those studies with the same anthracycline and taxane chemotherapy backbone in both arms were eligible.

Information on study design characteristics of patients enrolled, treatment received and study results were collected.

### 4.5. Quality of Evidence Evaluation

According to the GRADE approach, an evaluation of the certainty of evidence for each selected outcome was performed. The GRADE evaluation encompasses five main domains: study limitations, imprecision, indirectness, inconsistency and publication bias. Based on the study design, the certainty level starts at a pre-specified level (high certainty for randomized controlled trials). The detection of limitations in one or more of the five domains can lead to downgrading the certainty of evidence. The final judgment can be one of the following: high, moderate, low and very low. A summary of the certainty of evidence and a quantitative synthesis of the effects for each outcome are reported in a dedicated evidence profile table.

### 4.6. Evidence to Decision (EtD) Framework

The EtD framework provides a transparent and structured approach to support the decision-making process [36]. It allows to summarize the evidence in relation to the priority of the problem, the substantiality of the desirable and undesirable effects, balance of the effects, certainty of evidence, patients’ values and preference, use of resources, equity, acceptability, and feasibility.

### 4.7. Benefit/Harm Balance and Clinical Recommendation

The panel voted one of the following option for the balance between benefits and harms of the intervention and the comparison: favorable, uncertain/favorable, uncertain/unfavorable, and unfavorable. The panel also voted the strength of the recommendation according to the following options: strong positive, conditional positive, conditional negative, strong negative.

The AGREE-reporting checklist was followed to guide the reporting of the recommendation [37].

## 5. Conclusions

In conclusion, with the available evidence, the AIOM panel defined the inclusion of platinum in NACT for TNBC as an option. The strength of recommendation “conditional positive” indicates that there is still a not-negligible uncertainty related to the effect of the intervention. Therefore, the individual decision should be based on case-by-case clinical reasoning [38], balancing benefits, harms, and patient’s preferences. Further follow-up might clarify the long-term impact of platinum-containing regimens.

## Figures and Tables

**Table 1:** Main characteristics of the studies considered for the GRADE assessment of the clinical question.

*Study Name*	*Phase*	*n TNBC*	*Treatment Arms*
*BrighTNess* [15] *	*III*	*338*	*P 80 mg/mq qw for 12 w + Cb AUC 6 q3w for 4 courses → AC (60 mg/mq and 600 mg/mq) q3w for 4 courses* *P 80 mg/mq qw for 12 w + Cb AUC 6 q3w for 4 courses → AC (60 mg/mq and 600 mg/mq) q3w for 4 courses*
***CALGB 40603* [16] ^§^**	*II*	*454*	*P 80 mg/mq qw for 12 w + Cb AUC6 q3w for 4 courses +/− Bev 10 mg/kg q2w for 9 courses° → ddAC (60 mg/mq and 600 mg/mq q2w for 4 courses, with myeloid growth factor support)* *P 80 mg/mq qw for 12 w +/− Bev 10 mg/kg q2w for 9 courses° → ddAC (60 mg/mq and 600 mg/mq q2w for 4 courses, with myeloid growth factor support)*
***GeparSixto GBG66* [17] ^**	*II*	*315*	*P 80 mg/mq qw + nplD 220 mg/mq qw + Bev 15 mg/kg q3w + Cb AUC 2.0 or 1.5 qw for 18 w* *P 80 mg/mq qw + nplD 220 mg/mq qw + Bev 15 mg/kg q3w for 18 w*
***GEICAM/2006-3* [18]**	*II*	*93*	*EC (90 mg/mq and 600 mg/mq q3w for 4 courses) → Doc 75 mg/mq q3w for 4 courses + Cb AUC6 q3w for 4 courses* *EC (90 mg/mq and 600 mg/mq q3w for 4 courses) → Doc 100 mg/mq q3w for 4 courses*
***UMIN000003355* [19] ^**	*II*	*75*	*P 80 mg/mq qw for 12 w + Cb AUC5 q3w for 4 courses → CEF (500 mg/mq, 100 mg/mq and 500 mg/mq) q3w for 4 courses* *P 80 mg/mq qw for 12 w → CEF (500 mg/mq, 100 mg/mq and 500 mg/mq) q3w for 4 courses*

Abbreviations: n, number; TNBC, triple negative breast cancer; P, paclitaxel; Cb, carboplatin; AC, doxorubicin and cyclophosphamide; Bev, bevacizumab; dd, dose dense; nplD, non-pegylated liposomal doxorubicin; EC, epirubicin and cyclophosphamide; Doc, docetaxel; mq, square meter; AUC, area under the curve; qw, every week; w, week; q3w, every 3 weeks; * Arm with veliparib not considered; § For the analysis of the outcomes of harm only treatment arms not containing bevacizumab were considered; ° Bevacizumab was administered concurrently to paclitaxel and the first 3 courses of doxorubicin/cyclophosphamide; ^ For the analysis of the outcomes of harm, data in the entire patient population (included non-TNBC) were considered.

**Table 2:** Evidence profile table for GRADE assessment. **Question**: In patients with triple-negative breast cancer who are candidates to receive neoadjuvant chemotherapy, is the addition of a platinum agent to a taxane and anthracycline-containing regimen recommended versus a taxane and anthracycline-containing regimen only? **Setting**: inpatients. **Bibliography**: BrighTness [15], CALGB 40603 Alliance [16,20], GeparSixto GBG66 [17,21,22], GEICAM/2006-03 [18], and UMIN000003355 [19].

Certainty Assessment							No of Patients		Effect		Certainty	Importance
№ of studies	Study Design	Risk of Bias	Inconsistency	Indirectness	Imprecision	Other Considerations	Platinum Added to Taxane- and Anthracycline-Based Neoadjuvant Chemotherapy	Taxane- and Anthracycline-Based Neoadjuvant Chemotherapy only	Relative (95% CI)	Absolute (95% CI)		
**Overall survival (follow up: range 39 months to 47.3 months)**												
3 ^a^	randomised trials	not serious	serious ^b^	not serious	serious ^c^	none ^d^	33/384 (8.6%) ^e^	36/385 (9.4%) ^e^	**HR 0.86** (0.46 to 1.63)	**1 fewer per 100** (from 5 fewer to 5 more)	⨁⨁◯ LOW	CRITICAL
**DFS/EFS (follow up: range 39 months to 47.3 months)**												
4 ^f^	randomised trials	serious ^g^	serious ^h^	not serious	serious ^i^	publication bias STRONGLY suspected ^j^	75/384 (19.5%) ^e^	100/385 (26.0%) ^e^	**HR 0.72** (0.49 to 1.06)	**7 fewer per 100** (from 12 fewer to 1 more)	⨁◯◯◯ VERY LOW	CRITICAL
**Pathological complete response rate (assessed with: no residual invasive tumour in both the breast and the axilla: i.e., ypT0/is pN0)**												
5 ^k^	randomised trials	serious ^l^	serious ^m^	not serious	not serious	none	338/623 (54.3%) ^e^	229/611 (37.5%) ^e^	**RR 1.45** (1.28 to 1.64)	**17 more per 100** (from 10 more to 24 more)	⨁⨁◯◯ LOW	CRITICAL
**Febrile neutropenia**												
5 ^k^	randomised trials	not serious	not serious	not serious ^n,o^	serious ^i^	none	63/701 (9.0%)	44/695 (6.3%)	**RR 1.42** (0.98 to 2.06)	**3 more per 100** (from 0 fewer to 7 more)	⨁⨁⨁◯ MODERATE	CRITICAL
**Anemia grade 3/4**												
5 ^k^	randomised trials	not serious	not serious	not serious ^n,o^	serious ^i^	none	99/701 (14.1%)	2/695 (0.3%)	**RR 49.08** (12.15 to 198.20)	**14 more per 100** (from 3 more to 57 more)	⨁⨁⨁◯ MODERATE	IMPORTANT
**Seriuos adverse events**												
3 ^p^	randomised trials	serious ^q^	not serious ^r^	not serious ^n,^^o^	not serious	none	174/566 (30.7%)	134/558 (24.0%)	**RR 1.28** (1.06 to 1.55)	**7 more per 100** (from 1 more to 13 more)	⨁⨁⨁◯ MODERATE	CRITICAL
**Thrombocytopenia grade 3/4**												
5 ^k^	randomised trials	not serious	not serious ^s^	not serious ^n,o^	serious ^i^	none	79/701 (11.3%)	5/695 (0.8%)	**RR 15.66** (6.38 to 38.44)	**11 more per 100** (from 4 more to 27 more)	⨁⨁⨁◯ MODERATE	IMPORTANT

**CI:** confidence interval; **HR:** hazard ratio; **RR:** risk ratio; **Explanations**: a. CALGB 40603 Alliance, GeparSixto GBG66 and BrighTness. b. I-squared = 63.9%. c. Downgraded for imprecision due to the small number of events. d. Only two reported survival analysis. OS was a secondary endpoint for BrighTNess, but data have not been reported yet. Follow-up was probably too short for survival analysis; therefore, no publication bias was suspected. e. CALGB 40603 Alliance: intention-to-treat population considered (all randomized). f. CALGB 40603 Alliance, GeparSixto GBG66, UMIN000003355 and BrighTNess. g. Possible detection bias due to the lack of masking in GeparSixto. h. I-squared = 33%. i. Downgraded for imprecision due to the small number of events. j. UMIN000003355 and BrighTNess studies stated as secondary outcome DFS/EFS but did not report data. Follow-up was probably too short for survival analysis; therefore, no publication bias was suspected. Instead, follow up in UMIN000003355 may be mature for survival analysis, therefore, a publication bias was suspected. k. CALGB 40603 Alliance, GeparSixto GBG66, BrighTness, GEICAM/2006-03 and UMIN000003355. l. Possible detection bias due to the lack of blinded outcome assessor. m. I-squared = 55%. n. For BrighTNess study, safety data occurring during the first segment of neoadjuvant chemotherapy were considered. o. UMIN000003355 and GeparSixto studies reported in the entire study population, including non-TNBC. HER2-positive patients in GeparSixto GBG66 also received trastuzumab and lapatinib. p. GeparSixto GBG66, BrighTness and CALGB 40603 Alliance. q. Possible detection bias due to the lack of masking in CALGB 40603 and GeparSixto GBG66 studies. r. I-squared = 26%, s. I-squared = 16%.

**Table 3:** Final recommendation and summary of GRADE evaluation.

**CLINICAL QUESTION: In patients with triple-negative breast cancer who were to receive neoadjuvant chemotherapy, was the addition of a platinum agent to a taxane and anthracycline-containing regimen recommendable versus a taxane and anthracycline-containing regimen only?**			
**Recommendation:** In patients with triple-negative breast cancer, receiving neoadjuvant chemotherapy, the addition of platinum to a standard regimen containing anthracycline and taxane may be taken into consideration			
**Strength of recommendation:** CONDITIONAL POSITIVE			
**Quality of evidence: Outcomes of benefit: Very Low; Outcomes of harm: Moderate**			
**Reasons/comments on the benefit/harm balance** **Outcomes of benefit.** The Panel identified as “critical” the following outcomes of benefit: pCR, DFS/EFS and OS. Five randomized controlled trials (RCTs) with the same anthracycline and taxane-based chemotherapy backbone in the platinum (carboplatin) and no-platinum arms were considered for the assessment of the clinical question [17,18,19,20,21] All five studies reported pCR [17,18,19,20,21] and only two studies reported survival data [18,19,22,23,24]. The risk of pCR was 54 every 100 patients with carboplatin and 37 every 100 patients without carboplatin (RR 1.45, 95% CI 1.28–1.64). The quality of evidence in support of pCR outcome was low (potential bias due to the possible lack of blinded outcome assessor and heterogeneity with I-squared 55%). There was no significant difference in favor of the addition of platinum for: DFS/EFS (HR 0.72, 95% CI 0.49–1.06) and OS (HR 0.86, 95%CI 0.46–1.63). Although the available evidence was insufficient to detect a significant difference in survival (low number of events and short follow-up), considering the difference in pCR, the panel judged as “moderate” the substantiality of the desirable effects derived from the addition of platinum to anthracycline and taxane-based neoadjuvant chemotherapy. **Outcomes of harm.** The Panel identified the following outcomes of harms: febrile neutropenia, anemia (grade 3–4), serious adverse event (SAE), thrombocytopenia (grade 3–4). Only febrile neutropenia and SAE were considered “critical outcomes”. The addition of platinum to a standard anthracycline and taxane-based chemotherapy was not associated with a significant difference in the risk of febrile neutropenia (RR 1.42, 95% CI 0.98 to 2.06). The addition of platinum significantly increased the risk of SAE (RR 1.28, 95% CI 1.06 to 1.55), grade 3–4 anemia (RR 49.08, 95% CI 12.15 to 198.20), and grade 3–4 thrombocytopenia (RR 15.66, 95% CI 6.38 to 38.44). The panel also considered the adverse events occurring in the BrighTNess study in the second treatment segment (anthracycline-based) that were not included in the overall assessment. The Panel, considering the heterogeneity of treatment schedules applied in the different studies that might have influenced the incidence of adverse events, judged as “small” the substantiality of the undesirable effects. Although the survival impact of the addition of platinum remains undefined, the Panel voted for the benefit/harm balance as uncertain-favorable, considering the increase in pCR rates and the acceptable safety costs.			
**Vote on benefit/harm balance**			
**Favorable**	**Uncertain (Favorable)**	**Uncertain (Unfavorable)**	**Unfavorable**
	**10**	**1**	
**Vote on strength of recommendation**			
**Strong Positive**	**Weak Positive**	**Weak Negative**	**Strong Negative**
	**10**	**1**	
**Implications for future research:** a longer follow-up of those studies that report DFS/EFS and/or OS as secondary endpoint is necessary in order to define the survival impact of the addition of platinum.			

## Appendix A. Research Strategy

PubMed

((((((((((((((“Triple Negative Breast Neoplasms/drug therapy” [Mesh] OR “Triple Negative Breast Neoplasms/pathology” [Mesh]))) OR (“triple negative breast Cancer” [Title/Abstract] OR “triple negative breast neoplasms” [Title/Abstract] OR “triple negative breast tumo*” [Title/Abstract]))))))))

AND

((((((((((((((((“Platinum Compounds/therapeutic use” [Mesh]) OR ((((((((“Carboplatin” [Mesh]) OR “Platinum” [Mesh])) OR (Cisplatinum [Text Word] OR carboplatinum [Text Word] OR Platinum [Text Word] OR “platinum compound*” [Text Word] OR “platinum containing regime*” [Text Word] OR biocisplatinum [Text Word] OR platino [Text Word] OR platinol [Text Word] OR paraplatin [Text Word])) OR (((cisplatin [Text Word] OR oxilaplatin [Text Word] OR carboplatin [Text Word]))))))) OR (“Neoadjuvant Therapy” [Mesh]) OR “Neoadjuvant Therapy” [Title/Abstract]))))))

AND

((((“Neoadjuvant Therapy” [Mesh]) OR “Neoadjuvant Therapy” [Title/Abstract])))))) OR ((“Anthracyclines” [Mesh]) OR (Anthracycline* [Text Word] OR Daunorubicin [Text Word] OR Doxorubicin [Text Word] OR Idarubicin [Text Word] OR epirubicin [Text Word] OR valrubicin [Text Word] OR Aclarubicin [Text Word] OR Carubicin [Text Word] OR Nogalamycin [Text Word] OR Plicamycin [Text Word])))) AND
((((((“Neoadjuvant Therapy” [Mesh]) OR “Neoadjuvant Therapy” [Title/Abstract])))))))) OR ((“Taxoids” [Mesh]) OR (Taxoids [Text Word] OR docetaxel [Text Word] OR paclitaxel [Text Word] OR nab-paclitaxel [Text Word] OR Cabazitaxel [Text Word]))))

Embase

#1 ‘triple negative breast cancer’/exp OR ‘triple negative breast cancer’
#2 ‘triple negative breast cancer’ OR ‘triple negative breast neoplasms’ OR ‘triple negative breast tumo*’:de,ti,ab
#3 #1 OR #2
#4 ‘platinum derivative’/exp OR ‘carboplatin’/exp
#5 cisplatinum OR carboplatinum OR platinum OR ‘platinum compound*’ OR ‘platinum containing regime*’ OR biocisplatinum OR platino OR platinol OR paraplatin OR cisplatin OR oxilaplatin OR carboplatin:de,ti,ab
#6 #4 OR #5
#7 ‘neoadjuvant therapy’/exp
#8 ‘neoadjuvant therapy’:de,ti,ab
#9 #7 OR #8
#10 ‘anthracycline’/exp
#11 anthracycline* OR daunorubicin OR doxorubicin OR idarubicin OR epirubicin OR valrubicin OR aclarubicin OR carubicin OR nogalamycin OR plicamycin:de,ti,ab
#12 #10 OR #11
#13 ‘taxoid’/exp
#14 taxoids OR docetaxel OR paclitaxel OR nab-paclitaxel OR cabazitaxel:de,ti,ab
#15 #13 OR #14
#16 #6 AND #12 AND #15
#17 #9 OR #16
#18 #3 AND #17
#19 ‘crossover procedure’:de OR ‘double-blind procedure’:de OR ‘randomized controlled trial’:de OR ‘single-blind procedure’:de OR random*:de,ab,ti OR factorial*:de,ab,ti OR crossover*:de,ab,ti OR ((cross NEXT/1 over*):de,ab,ti) OR placebo*:de,ab,ti OR ((doubl* NEAR/1 blind*):de,ab,ti) OR ((singl* NEAR/1 blind*):de,ab,ti) OR assign*:de,ab,ti OR allocat*:de,ab,ti OR volunteer*:de,ab,ti
#20 #18 AND #19
#21 #18 AND #19 AND ( [article]/lim OR [article in press]/lim) AND [embase]/lim

Cochrane

#1 MeSH descriptor: [Triple Negative Breast Neoplasms] explode all trees
#2 (‘triple negative breast cancer’ OR ‘triple negative breast neoplasms’ OR ‘triple negative breast tumo*’):ti,ab,kw (Word variations have been searched)
#3 #30 OR #31
#4 MeSH descriptor: [Platinum Compounds] explode all trees
#5 MeSH descriptor: [Carboplatin] explode all trees
#6 (cisplatinum OR carboplatinum OR platinum OR ‘platinum compound*’ OR ‘platinum containing regime*’ OR biocisplatinum OR platino OR platinol OR paraplatin OR cisplatin OR oxilaplatin OR carboplatin):ti,ab,kw (Word variations have been searched)
#7 #33 OR #34 OR #35
#8 MeSH descriptor: [Anthracyclines] explode all trees
#9 (anthracycline* OR daunorubicin OR doxorubicin OR idarubicin OR epirubicin OR valrubicin OR aclarubicin OR carubicin OR nogalamycin OR plicamycin):ti,ab,kw (Word variations have been searched)
#10 #37 OR #38
#11 MeSH descriptor: [Taxoids] explode all trees
#12 (taxoids OR docetaxel OR paclitaxel OR nab-paclitaxel OR cabazitaxel):ti,ab,kw (Word variations have been searched)
#13 #40 OR #41
#14 MeSH descriptor: [Neoadjuvant Therapy] explode all trees
#15 #36 AND #39 AND #42
#16 #44 OR #43
#17 #45 AND #32
